# Doing philosophy effectively II: A replication and elaboration of student learning in classroom teaching

**DOI:** 10.1371/journal.pone.0208128

**Published:** 2018-12-03

**Authors:** Natascha Kienstra, Peter G. M. van der Heijden

**Affiliations:** 1 Tilburg School of Catholic Theology, Tilburg University, Utrecht, the Netherlands; 2 Radboud Teachers Academy, Radboud University, Nijmegen, the Netherlands; 3 Department of Methodology and Statistics, Utrecht University, Utrecht, the Netherlands; 4 S3RI, University of Southampton, Southampton, United Kingdom; The University of New England, AUSTRALIA

## Abstract

An important aim of teaching philosophy in Dutch secondary schools is to learn about philosophy (that is, the great philosophers) by doing philosophy. In an earlier study published in *PLoS ONE*, we focused on the relationship between student learning activities and teacher behavior by analyzing eight lessons. Correspondence analysis revealed that doing philosophy was more effective in some lessons than in others. We replicated this finding in the current study, using 10 new lessons, and elaborated on the relationship between the likely causes for doing philosophy effectively. The data suggest that conducting a dialogue in the form of a philosophical discussion is sufficient for achieving an effective lesson, whereas the teachers’ guidance being shared with the students is a necessary but not sufficient condition for achieving an effective lesson.

## Introduction

An important aim of teaching philosophy in Dutch secondary schools is to learn about philosophy (i.e., the great philosophers’ contributions to the world, in terms of civilization’s understanding of the nature of knowledge, reality, and existence) by doing philosophy. Constructivism is the learning theory that posits students “learn by doing” [1]. Learning, then, is seen as a largely interactive process of constructing new knowledge and skills based on the information that a person already has [2]. The phrase “doing philosophy” was used by Rudisill to describe students’ abilities to engage in philosophical activities. Thus, doing philosophy is in contrast to just being competent with a specific domain of knowledge [3]. In an earlier study reported in *PLoS ONE*, we examined doing philosophy by analyzing eight lessons. We focused specifically on the relationships between students’ learning activities and teachers’ behaviors, while also investigating the correlates of doing philosophy effectively [4]. Correspondence analysis (CA) revealed that these eight lessons were ordered on a scale from lessons where doing philosophy was more effective to those when it was less effective. As the number of cases in the earlier study was small, the stability of the CA solution was investigated by omitting variables and cases. We concluded that the CA solution was very stable under these modifications of the data.

We will now report on new data that were collected in the first measurement of an intervention study, in which teachers developed teaching materials according to prescribed design principles, and tested the material in their own teaching practice. We kept in touch with the teachers throughout the study period, and observed and recorded two of their philosophy lessons, one at the beginning of the intervention and one at the end. The first lessons are comparable to the lessons in the earlier study, and are analyzed here to investigate whether the findings of the earlier study can be replicated. Second, we elaborated on the relationship between the likely causes for doing philosophy effectively, namely choosing a dialogue in the form of a philosophical discussion in the classroom teaching, where the teachers’ form of guidance is shared with the students. We used the causal complexity methodology developed by Ragin (see chapter 4 in [5])4, to test whether two different conditions for doing philosophy were either necessary or sufficient. These two conditions were philosophical discussions and shared guidance.

### Theoretical framework and earlier results

Unlike schools in other countries, in the Netherlands, secondary schools can choose to include philosophy as a distinct, optional, secondary school subject from the tenth grade onward for pre-university and senior general higher education students [6]. Teachers must have a Master’s in Philosophy, after which they study philosophy pedagogy for their education degrees. A number of substantive philosophical domains are used to teach philosophy at secondary schools in the Netherlands [7, 8] (e.g., philosophical anthropology, ethics, social philosophy, theory of knowledge, and the philosophy of science).

How do students learn to do philosophy by themselves during a philosophy lesson? To answer this question, we developed a conceptual schedule for such a lesson (see Fig 1 in [4]). The relationship between teaching behavior and students’ doing philosophy is influenced by the teacher’s lesson design. Lesson design, teacher behavior, and students doing philosophy correspond with concepts from curriculum research [9, 10], which speaks of a curriculum characterized by intention (lesson design), implementation (teaching behavior), and attainment (students doing philosophy). A similar threefold division was also noted by Imants [11], who spoke of design, execution, learning activities, and the results of learning.

#### Students doing philosophy

**Fig 1:**
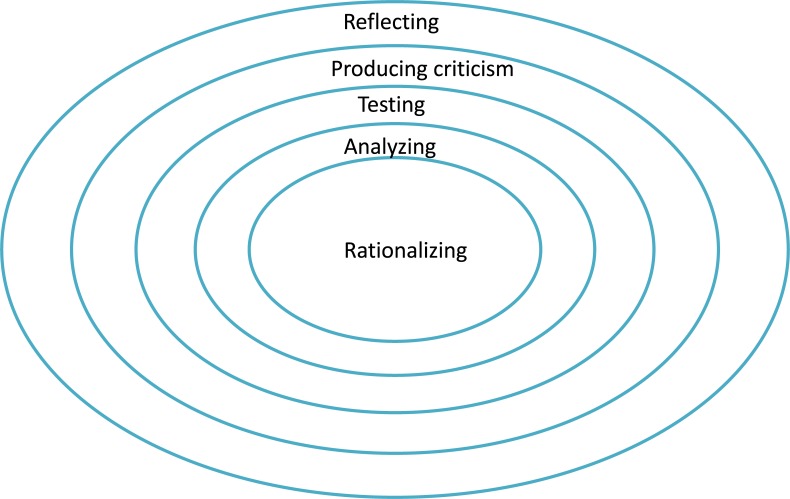
Pearl model.

What behaviors do students actually engage in when they do philosophy? We developed the Pearl Model of doing philosophy in the classroom [4] to characterize such moments (see Fig 1). It enables us to assess the different levels of complexity involved in doing philosophy, from low to high, as follows: rationalizing, analyzing, testing, production of criticism, and reflecting [12]. *Rationalizing* involves the verbalization of initial thoughts in a logical structure, while *analyzing* entails continuous questioning, wondering, interrogation, problematization, and consideration. *Testing* encompasses evaluation, establishing definitions and distinctions, and making judgments. The *production of criticism* involves reasoning based on explanations, causes, and connections, in addition to pro/con arguments, the construction and maintenance of logical arguments, and debate. Finally, *reflection* entails making metaremarks, mirroring, creative leaps, and thinking about the thought process itself, as well as reflecting on pro/con arguments, the assessment framework, and its application [4]. In the Pearl Model, pearls are composed of concentric layers that represent each of the five aforementioned activities. These activities are ordered hierarchically and conditionally. This indicates, for example, that while rationalizing exists at a lower level than reflecting, reaching the level of reflection assumes that rationalizing has also has taken place. Therefore, the higher the level that a pearl reached and the more layers have been reached, the more thorough the philosophical understanding, and the more effectiveness of doing philosophy are [4]. Thus reflecting is the highest level of doing philosophy.

An important part of doing philosophy is having students construct a common concept (i.e., an answer) to a philosophical question [13]. While doing philosophy, students make abstract words more concrete (deductive ladder). For example, with regard to happiness, a student can say, “When I was 16 years old and was on holiday abroad…”. Another example is to think aloud about how a concept can be used substantively (sentence building), such as “happiness can shake your soul”, or create definitions (defining), as in “happiness is…”, and search for counterexamples and explore boundaries (counterexamples and exploring boundaries), as for example “misery and joy” [14].

In our earlier study, using CA, we found that lessons were scalable from less to more effective. In the more effective lessons, the more often the highest layer of pearl (reflecting) was reached, the larger the number of pearls (4–6) found, the larger the percentage of time spent on them in the lesson, and the more use was made of building sentences, proposing counterexamples, and exploring boundaries [4].

#### Teacher behavior

Teacher behavior in the philosophy classroom is concerned with (i) philosophical teaching styles, (ii) the form of dialogue, and (iii) the form of guidance [4].

*Philosophical teaching styles*. According to the problem-oriented teaching style, doing philosophy means solving philosophical problems or finding answers to philosophical questions. According to the historical teaching style, the most important task of the philosopher is interpreting and reinterpreting the philosophical past using existing philosophical texts. According to the person-oriented teaching style, doing philosophy is an attempt to create an individual, reasonably justified worldview. Another conception of philosophy posits that there are greater consequences for a teacher’s practice than telling another story or reading other texts in the classroom, because it implies a completely different atmosphere in the classroom, with another distribution of roles for the teacher and the students. In one classroom, students work quietly on a problem while the teacher assigns them tasks; in another class the teacher provides a lively performance while fascinated students listen, and in the third there may be a lively exchange of ideas ([15], p. 24, authors’ translation).

*Form of dialogue*. Philosophy teachers can do philosophy with students in either an open or a more closed form [16]. A dialogue in the form of a philosophical discussion is a more open form than a classroom talk. A classroom talk is always (re)directed by the instructor. In a philosophical discussion, students engage critically but constructively with each other’s ideas. Positions and suggestions are proposed, challenged, and defended, but the contestations are reasonable, and alternative hypotheses are offered. All students take an active part in the discussion, and opinions are examined before any judgments are made. This type of talk resembles Mercer and Littleton’s exploratory talk [17], which has not only a descriptive purpose but also an evaluative dimension, and gives attention to effectiveness.

*Form of guidance*. An important aspect of process-oriented teaching is its focus on the gradual transfer of the teacher’s thinking and learning processes to the student. Guidance can be strong, shared, or loose [18, 19]. Strong guidance occurs when it is mostly the teacher who determines the content of the conversation during the lesson. If the teacher pulls back from the discussion and the students determine what the conversation is about, this is described as loose guidance. When a teacher and the students engage in a common dialogue, and jointly determine the content, this is shared guidance.

After we collected and scored the observational data from the current study, contributions from teachers were characterized as student-regulative when these contributions were focused on guiding the discussion process without regulating the content of the discussion, as co-regulative when teachers used the students’ perspectives and ideas to regulate the discussion, and as teacher-regulative when the teacher predominantly determined and directed the content of the discussion, as for example, by bringing in new content or by discouraging or curtailing certain perspectives put forward by students (for details, see [20]). We had the impression that these three terms referred to the same processes as our three types of guidance, and, as our current study aims to replicate it [4], we retained the terminology from that study.

In our earlier study we found a greater variety of different philosophical teaching styles in more effective philosophy lessons (although it should be noted that there was only one lesson using more than one teaching style). It also appeared that students more often do philosophy effectively during philosophical discussions than during classroom talks. The reason for this is probably that students involved in a discussion tend to do philosophy together (they think aloud about how a concept can be used substantively, they work on concept formation, search for counterexamples, and explore boundaries). Furthermore, doing philosophy effectively was more likely to occur with shared guidance than with loose or strong guidance [4].

#### Lesson design

In the context of doing philosophy in the classroom, Kienstra et al. [12] identified three forms of truth finding:

1. Doing philosophy as a method of connective truth finding or communicative action
2. Doing philosophy as a type of test-based truth finding
3. Doing philosophy in the form of a juridical debate, which entails judging truth-value and making judgments (i.e., truth-value analysis)

The first form involves doing philosophy as a form of connective truth finding, wherein students search for the truth together using narratives and conversations. The second is doing philosophy as a kind of test-based truth finding, in which students search for scientific truths, as practiced by scientists. The third is doing philosophy as a juridical way of finding the truth and truth-value of the competing/different or opposite claims through analysis by a competent judge and reaching a “verdict” [21]. In our research, we present these three forms of doing philosophy as a relevant educational context in which teacher and student activities can be understood.

In our earlier study, the juridical debate approach was observed more often in more effective lessons; the approach of connective truth finding was observed more often in less effective lessons; and the test-based truth-finding approach was found in both more and less effective philosophical lessons [4].

The purpose of this article is to replicate the previous research into doing philosophy effectively. In the previous research, CA yielded a dominant one-dimensional structure in the data, and therefore attention could be focused on the interpretation of this dimension, and lessons could be ordered by level of effectiveness in terms of doing philosophy. This leads to the following research question:

RQ1: Can the one-dimensional structure found in the earlier study using CA be replicated?

A second question is:

RQ2: Can we use the data from the earlier study and the new data to obtain a better understanding of how teacher behavior influences the level of doing philosophy?

## Methods

As in the earlier study, we observed, analyzed, and compared the participating teachers’ lessons using a mixed-methods approach—which includes both qualitative and quantitative research methods—for the empirical aspect of this research [22]. We used qualitative comparative case analysis (cross-case synthesis [23]) of lessons from different teachers for the data collection phase, to increase the generalizability to cases (lessons from other teachers) that were not observed [4]. In the data collection phase, the interpretation of the findings will lead to separate case-wise summaries in a matrix of mostly qualitative findings. These case-wise results were subsequently summarized in a matrix described by Miles and Huberman [24] as a “meta-matrix.”

The meta-matrix was studied using a quantitative methodological approach called correspondence analysis (CA) [25], which allows a quantitative comparative analysis of cases using graphical multidimensional (most often one- or two-dimensional) representations. In our earlier study [4], this method yielded a very stable first dimension, even though the number of cases studied was relatively small (eight).

### Participants

We observed nine philosophy teachers—four females and five males, all of which held a Master’s in Education from one of five different university teacher training programs in the Netherlands—teach ten lessons to their students. We studied one lesson from each of eight teachers, and two lessons from one teacher. All nine teachers participated in a project to develop Dutch philosophy textbooks to be used in secondary education, and our lesson observations were made at the beginning of the intervention. Three teachers had participated in the earlier study [4]. A tenth teacher dropped out of the project and did not provide any lessons. We considered analyzing only one lesson from the teacher who provided two lessons, but chose not to do so, as reducing the number of lessons from ten to nine represented a loss of precious data (the final analysis was verified by eliminating each of the two lessons separately, which in both cases led to minor changes that did not lead to a change in the interpretation).

The selection criteria for this sample were pragmatic (including an active recruitment of female teachers) and strategic (country-wide professional coverage). Two teachers took part per thematic domain. As much as possible, we allowed the teachers to choose a thematic domain (e.g., philosophical anthropology, ethics, social philosophy, theory of knowledge, or philosophy of science), and they were asked to teach a lesson in that domain. They chose the exercise used in their lessons.

Prior to the lesson, each teacher was asked if during his or her lessons the teaching philosophy’s goals (i.e., to learn philosophy by doing philosophy) were usually achieved (“yes” or “no”) [26, 27]. Furthermore, the teachers’ background characteristics were collected, including whether the teacher had a Master’s in Philosophy [28], the years of teaching experience he or she had accumulated since obtaining a degree in teaching philosophy [29] (three responded “1–5 years,” one “6–10 years,” and five “11–15 years”), and whether the class in which the lesson was observed was a class of tenth graders (pre-university or senior general higher education) (seven times), eleventh graders (twice), or both (once).

In 2013, when the data were collected, there was no ethical review board in the department of philosophy, or in the teachers’ academy, from which we could seek approval for our research. Teachers were given a verbal overview of the study prior to beginning the project, and we obtained permission via email to videotape one of each instructor’s standard lessons; teachers were also provided with information to assist them in preparing their recordings. Appointments were later made with the teachers to record the lessons, after which the teachers distributed consent forms to the students for their parents to sign. The forms explained the study’s aim, and informed parents that their child would be videotaped. In cases where parents did not provide consent, the camera was positioned to ensure that their child would not be recorded. The lesson environment and content were not altered in any way for purposes of the study, and no personal details (e.g., information pertaining to physical or mental health) were collected.

### Data collection and approach

The data collection for this study was conducted similarly to that in the earlier study. The data sources were: the philosophy lessons taught, the teaching material, the exercise(s) used, and the teachers and their students’ reflections. For each lesson, data were collected using five instruments: (i) a short list with factual questions that was distributed to teachers prior to the lesson (see S1 File); (ii) classroom recordings and transcripts of interactions between teachers and students; (iii) a lesson observation in the form of a qualitative graphical time registration (see Fig 3 in [4]); (iv) short post-lesson questionnaires for all students present; and (v) recordings and transcripts of stimulated recall interviews with teachers conducted after class.

During the lesson our camera was either at the front of the class (facing the students), or at the center window (facing the students and their teacher). Practical matters such as the placement of electrical sockets and whether the teacher needed his or her desk dictated where the cameras were placed.

Though there has been a five-year time lag between collecting data and publishing the results of our data analyses in this manuscript, these results—which were published in Dutch in book form in December 2016 but also have relevance for international audiences—are now being published in English.

### Instruments

Data for each lesson were summarized in a table with descriptive information. All five instruments used for data collection provide information about three substantive themes in every philosophy lesson: (a) lesson design, (b) teacher behavior, and (c) students doing philosophy.

The results of the initial analysis are explained below, particularly the operationalizations used to design the lesson, the teacher behavior, and how the students did philosophy, and summarized in a coding scheme. The operationalizations are explained only briefly, because they have been discussed previously (see Table 1 in [4] for the coding scheme).

#### Lesson design

Lesson observations, post-lesson short questionnaires for all students present, and interviews with teachers conducted after the class were used to code approaches to doing philosophy into variables for the lesson design. The approaches used included connective truth finding (Ctf), test-based truth finding (Ttf), and juridical debate (Jd). The students were asked to “Please indicate the row that best suits the exercise that was used today in the lesson (circle A or B or C): A. narrative–own thoughts–constructing–connecting; B. science–logical structure–researching–testing; C. debate–argumentative structure–achieving a final judgment–reaching a verdict.” As reported in earlier research [4], three aspects were investigated in relation to these approaches for each lesson: design, execution, and learning activities. As in [4], in the super-indicator matrix (see S3 File), the numbers of different approaches used in each lesson are added together, to increase the stability of the CA solution.

#### Teacher behavior

As in our earlier study [4], we distinguished between philosophical teaching styles, the form of the dialogue, and guidance provided during the lesson. The form of dialogue used was coded under one of two categories, “philosophical discussion” and “classroom talk.” Substantive guidance provided by the teacher to the students was coded as “strong,” “shared,” or “loose.”

To gain insight into the teaching styles used by the instructors, the lesson observations, recorded lessons, and transcripts were analyzed to determine how often the three philosophical teaching styles—the historical, the problem-oriented, and the person-oriented—were actually combined (“1,” “2,” or “3”).

#### Students doing philosophy

As in earlier research [4], students doing philosophy was examined in different ways: By studying the number of pearls; the duration of the pearls (in seconds), and the percentage of the lesson duration spent on each pearl; the level of doing philosophy (i.e., the level of the pearl model) reached; and common concept formation using four methods (1. deductive ladder; 2. sentence building; 3. defining; and 4. counterexamples and exploring boundaries).

### Data quality and reliability

In qualitative research, steps must be taken to ensure data integrity. Here we present the results in terms of data quality and reliability. Our analysis of the 10 lessons observed generated 39 pearls. Thirty-eight pearls were written down (for teacher Aisha, the transcription of the first pearl was lacking, because the quality of the recording was too poor). Some of the teachers’ pearls—Marlies’s, Ramses’s, and John’s—could not be analyzed (1, 1, and 2 pearls, respectively), because the text was too fragmented. As a result, 34 pearls remained. Thus, in Table 1, the number of pearls is 39, but for the assessment of the highest level reached only 34 pearls were studied.

**Table 1:** Meta-matrix with results for variables in the context of design of a lesson, teacher behavior, and students doing philosophy for ten philosophy lessons.

VARIABLES		Lesson 1	Lesson 2	Lesson 3	Lesson 4	Lesson 5	Lesson 6	Lesson 7	Lesson 8	Lesson 9	Lesson 10
		*John 1*	*John 2*	*Jil*	*Oscar*	*Aisha*	*Marlies*	*Anna*	*Ramses*	*Ruud*	*Marc*
**I. Design of a lesson**											
*Approaches to doing philosophy*											
Connected truth finding (Ctf), Test-based truth finding (Ttf), Juridical debate (Jd)	Design	Ctf	Ctf	Ctf	Ttf	Ctf & Ttf	Ctf & Ttf	Ctf	Ctf	Ctf & Ttf	Ttf
	Execution	Ctf	Ctf & Jd	Ctf & Ttf	Ttf	Ctf	Ctf & Ttf	Ctf & Ttf	Ctf & Jd	Ctf	Ctf & Ttf
	Learning activities	Ctf	Ctf	Ctf	Jd	Ctf	Ctf	Ctf	Ctf	Ctf	Ttf
*Domains*		Social philosophy	Social philosophy	Philosoph anthropol	Ethics	Theory of knowledge	Theory of knowledge	Social philosophy	Ethics	Philosoph anthropol	Philosophy of science
*Teacher characteristics*											
Aim fulfilled		No	No	Yes	Yes	Yes	Yes	Yes	No	Yes	Yes
MA in philosophy		Yes	Yes	Yes	Yes	Yes	Yes	No	Yes	Yes	Yes
Years of experience after training (categorized)		1–5	1–5	1–5	11–15	6–10	11–15	11–15	1–5	11–15	11–15*
Number of teaching styles		2	2	2	2	3	3	2	2	2	2
*Student characteristics*											
Grade		10	10	10	10	11	11	10	10	10	10/11
**II. Teacher behavior**											
Interaction	Design of exercise	Classroom talk	Classroom talk	Classroom talk	Collaborative research	Socratic method	Brainstorming & classroom talk	Classroom talk	Class discussion	Socratic method	Induction game
	Execution of exercise (dialogue)	Classroom talk with strong guidance	Classroom talk with shared guidance	Classroom talk with shared guidance	Philosophical discussion with shared guidance	Classroom talk with loose/ shared guidance	Classroom talk with strong/ shared guidance	Classroom talk with shared guidance	Classroom talk with strong guidance	Philosophical discussion with shared guidance	Philosophical discussion with shared guidance
**III. Students doing philosophy**											
Number of pearls		3	3	5	4	3	4	4	4	3	6
Pearls (percentage of lesson)		21%	21%	24%	63%	10%	32%	60%	42%	41%	41%
Highest level reached (1–5)		4	4	4	5	3	4	4	3	5	5
Common concept formation in taxonomy of conceptual analysis **	Method 1	0%	0%	54%	14%	42%	30%	0%	0%	72%	0%
	Method 2	69%	40%	27%	67%	0%	20%	31%	67%	28%	0%
	Method 3	31%	19%	0%	0%	24%	0%	0%	8%	0%	9%
	Method 4	0%	41%	19%	19%	34%	50%	69%	26%	0%	91%

The classroom talk exercise was used in John’s philosophy lesson, which matches the criteria of the connective truth-finding approach. The exercise’s execution and learning activities were akin to the connective truth-finding approach. The substantive domain was social philosophy. The goal of learning philosophy by doing philosophy was unfulfilled. Oscar possesses a Master’s degree in philosophy and has 1–5 years of post-training teaching experience. He used two different philosophical teaching styles, and his students were tenth graders. The dialogue was a classroom talk with strong guidance. Three pearls were observed that spanned 21% of the lesson’s duration, and the fourth level (producing criticism) was the highest reached. Of the time pearls were observed, 69% and 31% utilized methods 2 (sentence building) and 3 (counterexamples/exploring boundaries), respectively.

*One-time retraining (allowed because of educational Master in subject other than philosophy): this took until September 2000 (interview was held in March 2013).

**Method 1 = deductive ladder; Method 2 = sentence building; Method 3 = defining; Method 4 = counterexamples/exploring boundaries.

In earlier research we presented interim results at a gathering of Dutch scholars in the field of philosophy teaching methods [4], who verified our interpretation and performed an independent assessment (they did not exchange information among themselves or with the researcher). The inter-rater reliability was 60 percent. We judged this as satisfactory, given that the raters were not familiar with the categories and were untrained in using the coding system.

As in the previous study [4], we conducted a stimulated recall-interview, and together with each teacher we identified the pearls of doing philosophy. This happened at each visit after class, through video recordings, transcripts, and observations. We then selected two pearls that we discussed in more detail during the interview (duration: approximately one hour). In doing so, we asked reflective questions for each pearl, in which the teacher in particular spoke out. Examples are: “Do you recognize my observations [that I saw today]?” “Do you do this more often?” “What is your side of the story here?” “Why did you do this, [and] why did you choose this?” “What went *wrong* and what went *well*?” “Where did the students make progress?” “Where was the progress the most significant?” “Please indicate the row that best suits the exercise that was used in the lesson today (circle “narrative–own thoughts–constructing–connecting,” or “science–logical structure–researching–testing,” or “debate–argumentative structure–achieving a final judgment–reaching a verdict”), and argue your choice.” “Please color this pearl (pearl model on a paper) and argue your choice. Please choose the *highest level* of doing philosophy reached, if multiple layers of the pearl could be colored.” “For this pearl, please mark one method of common concept formation that you find most appropriate, and argue your choice.” “Please designate one cell for this pearl (rows: strong/shared/loose guidance; columns: instructing/closed classroom talk/open classroom talk/philosophical discussion/class discussion/Socratic discussion), and argue your choice.” In addition, the researcher assessed the quality of the lesson during the lesson observation.

For each lesson, the highest level of pearls present was included in the meta-matrix. Therefore, the highest pearl evident in each lesson (as one of the two pearls) was discussed. In three cases the researcher’s and the teacher’s judgments were inconsistent, and in these instances colleagues were consulted to reach a decision: For teacher Ruud, another researcher provided the decisive argumentation (a student criticized the teacher with useful arguments; the fellow researcher gave this more weight, and reached a higher level). For teacher John, we gave more weight to our own interpretation (we felt that the teacher assessed *his own* activities rather than those of the students). This also happened for teacher Ramses (the teacher indicated *analyzing*, while in our view practical solutions were *tested* against values mentioned before). Of the 39 pearls observed, 19 were discussed with the teachers (three for John, who had two lessons, and two for each of the other teachers). In 16 of the 19 pearls, the researcher’s and teacher’s opinions were the same. The inter-rater reliability for teachers’ and researchers’ Pearl Model levels was 84 percent.

### Data

Similar to [4], for each lesson, the data are summarized in a meta-matrix (see Table 1). We provide reading instructions for John 1’s lesson, shown in the first column, for purposes of illustration. John’s philosophy lesson was based on learning activities in the connective truth-finding approach, and involved the classroom talk exercise. Its substantive domain was social philosophy. The goal of learning philosophy by doing philosophy remained unfulfilled.

Oscar had a Master’s in Philosophy and 1 to 5 years of post-training teaching experience. His students were tenth graders, and he used two different philosophical teaching styles. The dialogue used was a classroom talk with strong guidance. Three pearls that spanned 21 percent of the lesson’s duration were observed, and the fourth level (producing criticism) was the highest level reached. Of the pearls observed, 69 and 31 percent utilized methods 2 (sentence building) and 3 (counterexamples/exploring boundaries), respectively.

### Data analysis

#### Correspondence analysis (CA)

To compare individual philosophy lessons, the meta-matrix was analyzed with CA ([30], see also [4]) using the IBM SPSS-routine ANACOR (IBM SPSS input data, IBM SPSS syntax, and IBM SPSS output in S2 File). To analyze the meta-matrix using CA, it was first converted into a so-called super-indicator matrix (see S3 File, compare Table 3 in [4], which is provided here as S5 File). In this super-indicator matrix, 10 lessons are shown in 10 rows and the levels of the variables are listed in the columns. For example, the dialogue for teacher behavior was coded into three variables: one with teaching styles as levels, one with philosophical discussions and classroom talks as levels, and the other with types of guidance as levels. A lesson is assigned a value of 1 for the level it fell into, and a 0 for all other levels. Lesson 1, for example, was coded 1 for two teaching styles, classroom talk, and strong guidance. If a lesson fell into two levels for the same variable, however, it was assigned a value of .5 for each level (fuzzy coding) [4, 31].

As explained in more detail in [4], CA provides a graphical representation of the lessons, with those that are close in proximity indicating similarity and the presence of many levels of common variables. Likewise, columns (i.e., levels of variables) that are in close proximity denote the presence of similar variable levels, and thus indicate that they were used in similar lessons. Hence, the graphical representations of the levels (rows) and lessons (columns) are closely related. Apart from a scaling factor, the lessons are included in the means of the levels they used, and the levels are included in the means of the lessons in which they were used. The principles used to interpret CA are presented in S1 File and S4 File, taken from [4].

CA can be used to compare summaries of the case studies collected in a meta-matrix, which are coded into a super-indicator matrix. CA yields a remarkably stable solution when the number of cases is small ([4, 31], which covered only eight lessons). In these papers, stability was investigated by omitting two of the eight cases, and variables, and in none of the instances did this change the interpretation of the first solution in any relevant way. As the CA in [4, 31] was stable, it is expected that the CA in the current study of 10 lessons will be stable as well, and the two solutions in [4] can be meaningfully compared with the solution in the current study. In this comparison, we focus on the following: We first asked whether the first dimensions of the two solutions are mainly supported by the same variables, using the contributions of variables to the first solution. We then examined whether the levels of the most supportive variables in the two solutions are ordered in the same way along the first dimension, as this ordering of levels yields the interpretation of the first dimension.

It is well known that, in CA, levels of variables that are not used by a large proportion of the lessons may be placed far away from the other levels of variables [30]. In this instance, instead of giving insights into the larger picture of lessons and levels of variables, the solution reveals peculiarities in the data. This is a degenerate solution. To prevent degenerate solutions, unique coding was used for the levels most often shared by more than one lesson. Furthermore, in the current study, some levels [4] did not appear in the data. For these two reasons the levels used in the super-indicator matrix in the current study are slightly different from those used in the super-indicator matrix in [4].

The differences are as follows: (i) Experience after training was coded in [4] into levels 0 years, 1–5 years, 6–10 years, and 11–15 years, but in the current study it was coded into 1–10 years and 11–15 years; (ii) Student grades were coded in [4] into levels 10 and 11–12, but in the current study into 10 and 11 (because of the absence of classes having level 12); (iii) in [4] teaching styles included levels 1 and 3, but in the current study they are 1, 2, and 3; (iv) in [4] guidance was classified as strong, loose, and shared, but in the current study, loose hardly appeared (it was only featured in one half of a lesson), and so strong and loose were grouped together into one level; (v) in [4] the number of pearls was 2/3 and 4/5/6, but in the current study it was 1/2/3, 4, and 5/6/7; (vi) for duration in [4] we had low, middle, and high, but in the current study we chose low/mid, high, and very high. It should be noted that, although the levels of some of the variables changed, the meanings of the variables did not, as mostly only another order of levels came out. For details we refer to the meta-matrices and the super-indicator matrices in [4] and in the current study.

#### Focusing on the level of doing philosophy as a dependent variable

CA displays a matrix graphically. Underlying this graphic display is a set of all possible two-way contingency tables of the variables that are depicted in the graphic display. In this display, all variables play a symmetric role, in the sense that CA does not distinguish between dependent (response) and independent (predictor) variables. In the second part of the analysis we considered the level of doing philosophy a dependent variable. We studied the cases to find out how the responses regarding the level of doing philosophy varied as a result of variations in independent variables of teacher behavior—namely, dialogue and guidance.

This methodology is a simplified version of Ragin’s qualitative comparative analysis (QCA) [5], where multiple cases are studied from a comparative perspective. In Ragin’s work, the number of cases is usually larger than the number of cases at our disposal (eight cases from the earlier study and 10 from the current study), and therefore we can only apply it in a simple form. Ragin’s methodology is intended to enable researchers to develop causal statements from data using the binary logic of Boolean algebra. In this paper, we study the influence of the independent variables of teaching behavior in terms of the necessary and sufficient conditions needed to achieve higher levels of doing philosophy. The two methodologies, CA and qualitative comparative analysis, have been compared previously [32, 33]. In [32], in a simple situation, it was proposed that CA was complementary to QCA. Here we suggest the reverse order: CA was used to study a multitude of relations between variables, and this analysis generated ideas about relations between variables that we studied using QCA.

## Results

### Correspondence analysis (CA) solutions

**Fig 2:**
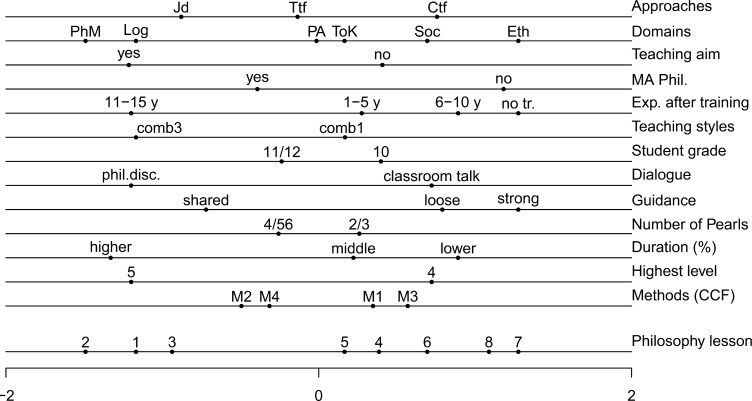
Correspondence analysis. Old solution taken from [4].

**Fig 3:**
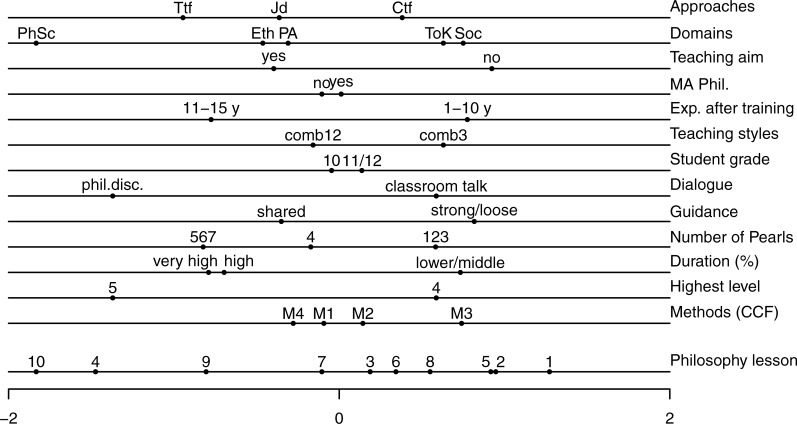
Correspondence analysis. Solution for current study.

The first dimension of the earlier study [4] is found in Fig 2, and the first dimension of the solution for the current study is shown in Fig 3. For ease of discussion, we refer to the CA solutions as the old solution (Fig 2, taken from [4]), and the new solution (Fig 3, current study). The variables’ contributions are displayed in Table 2. In our study of the new solution, we paid particular attention to whether the first dimension of CA scales the lessons in terms of whether they are more or less effective in doing philosophy. We also studied whether effectivity in the new solution is related to the same variables as in the old solution. We compared the old and the new solutions by checking two aspects of the solutions—namely, (i) whether the first dimension in both solutions is supported by the same variables, and (ii) whether for these variables the order of the categories in the old solution is identical to the order of the categories in the new solution. We noted that the low number of lessons studied, both in the old solution and in the new solution, could cause minor differences, and so we should be cautious of overinterpreting the findings.

**Table 2:** Contributions of the variables to the first dimension of the correspondence analysis solution.

	PONE 2015	Current study
Approaches	,144	,181
Domains	,086	,113
Teaching aims	,068	,067
MA Phil	,064	,000
Experience after training	,027	,018
Teaching styles	,133	,110
Student grade	,013	,001
Dialogue	,120	,147
Guidance	,095	,052
Number of Pearls	,010	,052
Duration (%)	,091	,098
Highest Level	,120	,147
Methods (CCF)	,025	,015

Aspect (i)—whether the first dimension in both solutions is supported by the same variables—is answered by the contributions made by the 13 variables (see Table 2). The first variable, Approaches, summarizes the three original variables mentioned in the operationalization of the lesson design: connective truth finding, test-based truth finding, and juridical debate. Therefore, the average contribution of variables, expressed as a proportion, is 1/15 = .067. In the old solution the first dimension was mainly supported by the variables teaching styles (.133), highest level and dialogue (.120), guidance (.095), duration (.091), domains (.086), and teaching aims (.068) (it should be noted that we do not mention approaches, which were .144, and .144/3 is smaller than .067). In the new solution we found the following variables: highest level and dialogue (.147), domains (.113), teaching styles (.110), duration (.098), and teaching aims (.067). These six variables in the new solution also contributed most in the old solution, where the only exception was guidance, which drops to .052 in the new solution. Interestingly, the number of pearls did not make a large contribution to either the new solution or the old solution.

For aspect (ii)—whether the order of the categories in the old solution is identical to the order of the categories in the new solution—we named the ordered categories from left to right:

- In both solutions, the highest level has the order 5, 4. This shows that the first dimension in the new solution can again be interpreted as an effectivity dimension. Dialogue has the order of philosophical discussion, classroom talk; duration has a comparable order in both solutions, in the old solution moving from higher, to middle, to lower, and in the new solution from very high, to high, to middle/lower; guidance has the order of shared, strong/loose in both solutions; teaching aims has the order of yes, and then no.
- We found different orders for domains and teaching styles, but it should be noted that the domains are not identical, and the number of domains used was large (five and six) in comparison to the number of lessons observed (eight and 10); the order of teaching styles was reversed from 3, 1 in the old solution to (1, 2), 3 in the new solution, but here again it should be noted that Category 3 is not used often (it appeared once in the old study and twice in the new study).
- The order for the approaches was partly different, where on the left the order of Jd and Ttf is reversed, but Ctf is on the right, meaning that it is least successful in both solutions.

We conclude that in large part, the new solution replicates the old solution as a dimension going from more successful lessons (on the left) to less successful lessons (on the right).

An extended discussion of the stability of CA was included in [4], since there were only eight lessons in that study. This study also had a small number of lessons, and it could be argued that the similarities of the CA solutions in [4] and the current study further confirm the stability of the solutions. However, for completeness, we also conducted stability analyses for the current study (see S2 File for input and output files). As in [4], we carried out 13 additional analyses with 12 variables, and removed a different one of the 13 variables each time. We then calculated the correlation between the original quantifications of the lessons (lowest line in Fig 3) and the quantifications of each of the 13 additional analyses. The correlations ranged from .974 to 1.000 (in [4] this was .992 to .999). We also studied the impact of removing the three variables that contributed most to the first dimension—Experience after training, Dialogue, and Highest level. For the remaining 10 variables, the correlation was still .869 (in [4] this was .955). A final way used to assess the stability was removing cases. As in [4], we excluded pairs of cases that were likely to be most influential (compare Fig 3): cases 1 and 10, cases 1 and 2, and cases 4 and 10. When we removed these pairs and calculated the correlations with the quantifications of the remaining 8 lessons in the full solution, we find correlations of .895, .942, and .538, respectively (in [4] these were .990, .988, and .981). By eliminating the two cases on the far left—namely, cases 4 and 10—the solution became unstable and this was a result of these two cases being so extreme. We therefore concluded that the solution in the current study was very stable.

To assess the effect of including only one of John’s two lessons, lessons 1 and 2, we conducted two analyses with nine lessons, one excluding lesson 1 and the other excluding lesson 2. We compared the correlations between the quantifications of the lessons for each of these solutions and the original solution, and verified correlations of .984 and .995, respectively, confirming that the solution is stable when one of John’s lessons is excluded, irrespective which one (see also S2 File).

### Focusing on the level of doing philosophy as a dependent variable

In Fig 1 of [4], the conceptual framework depicted a relationship between teacher behavior and students doing philosophy. In this study we treated the level of doing philosophy as the dependent variable. We focused on predicting the level of doing philosophy from two independent variables—namely, dialogue and guidance.

In both the 2015 study and the current study, dialogue accurately predicts the highest level variable: If the dialogue takes the form of a philosophical discussion, the highest level reached is reflecting (level 5), and for classroom talk the highest level reached is lower than reflecting (see Table 3). This suggests that a philosophical discussion is a sufficient condition for reaching the highest level of reflection. The role of guidance is more complicated for both studies as well: for shared guidance, we find level 5 or lower, but under loose/strong guidance level 5 is never reached. These results suggest that shared guidance is a necessary but not a sufficient condition for reaching level 5.

**Table 3:** Contingency tables showing the relations between the independent variables Guidance (upper tables) and Dialogue (lower tables) in the rows with the dependent variable Level of doing philosophy in the columns.

	**PONE 2015**		**Current study**	
	**Level 5**	**Lower**	**Level 5**	**Lower**
**Shared guidance**	Lessons 1,2,3	Lesson 5, half of 4	Lessons 4,9,10	Lessons 2,3,7, Half of 5/6
**Loose/strong Guidance**	None	Lessons 6,7,8 and half of 4	None	Lessons 1,8, Half of 5/6
	**PONE 2015**		**Current study**	
	**Level 5**	**Lower**	**Level 5**	**Lower**
**Philosophical discussion**	Lessons 1,2,3	None	Lessons 4,9,10	None
**Classroom talk**	None	Lessons 4,5,6,7,8	None	Lessons 1,2,3,5, 6,7,8

In Table 3, on Guidance the three cells that are filled appear to be ordered: at one extreme there is a cell where guidance is *not* shared, and the level of doing philosophy is lower than 5; at the other extreme there is a cell in which guidance is shared, and the level is 5; the third cell appears to fall in the middle, where there is shared guidance but level 5 is not yet reached. Thus in moving from a less effective lesson to an effective lesson, shared guidance is a first step towards doing philosophy effectively. It seems that a teacher, in moving from a less effective to a more effective lesson, must first be able to guide students in a shared way, and this will not always result in a lesson where level 5 is reached.

In Table 3, for the Current study on Guidance, the three cells that are filled can also be evident in Fig 3: lessons 4, 9, and 10 are clearly on the left in Fig 3; lessons 1, 8, and half of 5/6 are on the right; lessons 3, 7, and half 5/6 are in the middle. Lesson 2 is on the right in Fig 3, and not in the middle (as would be expected from Table 3), because it falls in categories of the other variables that are on the right, such as No for Teaching aim, Ctf for Approaches, Classroom talk for Dialogue, Lower/middle for Duration, and Lower for Highest Level. For the Current study on Dialogue, the lessons in the two cells are clearly separated in Fig 3.

## Discussion

The one-dimensional representations in Figs 2 and 3 are very similar, and the current study appears to replicate the results of the earlier study. This more detailed study of how teacher behavior predicts doing philosophy effectively appears to indicate that philosophical discussion is a sufficient condition for an effective lesson, while shared guidance is a necessary but not sufficient condition. The similarities between [4] and the current study are much greater than the dissimilarities.

With regard to the first research question, we focus first on the dissimilarities that deserve further study. The first issue is that both studies found that the approach connected to truth finding was least effective; in the earlier study juridical debate was more effective than test-based truth finding, while in the current study test-based truth finding is more effective than juridical debate. We note that 4 observations out of 24 in [4] and 3 out of 30 in the current study drew conclusions for juridical debate; therefore, more research is needed, possibly of a qualitative nature, before we can draw more definitive conclusions.

A second issue is that, although both studies found the third method, defining, to be the least effective, while the fourth method, counterexamples and exploring boundaries, was effective, both of the first and second methods, deductive ladder and sentence building, were unstable. In this case, then, a better understanding is needed before more definitive conclusions can be drawn.

A third issue is that, although in the earlier study the teachers with the most effective lessons all had a Master’s in Philosophy and in education, in the current study one of the teachers, Anna, did not have this background, although she did have a Master’s in Education, and provided an effective philosophy lesson. This indicates that having a Master’s in Philosophy is not a necessary condition for delivering an effective lesson.

With respect to the second research question, we must note a reservation by stating that philosophical discussion and shared guidance may be causally related to doing philosophy effectively. We have only observational data for this supposition, and so we can determine only relationships, not causes. In Fig 1 of [4], it was also assumed that teacher behaviors and students’ learning theoretically had a mutual relationship, and indeed, it may be that doing philosophy in a classroom effectively leads to shared guidance in a philosophical discussion, rather than the other way around; therefore, additional research is needed that allows for causal statements.

One may also wonder how it is possible that a philosophical discussion is sufficient for doing philosophy in a more effective way. We found that shared guidance was strongly related to doing philosophy effectively—namely, it is necessary but not sufficient. If a philosophical discussion is already sufficient, what is the further role of shared guidance? This answer is closely related to philosophical discussion and shared guidance, as can be derived from the meta-matrices. From a theoretical perspective, it is important to note that philosophical discussion is the form, while shared guidance deals with the content of the discussion. This theoretical perspective was provided in [12], where it was noted that a conventionally held belief is that doing philosophy for oneself does not suppose the accumulation of knowledge. However, a conversation about friendship shows a form of knowledge that can be deemed common sense. Therefore, doing philosophy requires a particular form of knowledge. In scientific philosophy, for example, common-sense knowledge appears to be insufficient, because it also seems to require understanding. Support for this notion is found in [34], p. 235n. Thus, in both the earlier and current studies, it appears that there is empirical support for the theoretical relation between form and content, that is, between philosophical discussion and shared guidance.

Finally, we made the following observation. Looking at the meta-matrices from both [4] and the current study, it appears that general exercises, in contrast to philosophical exercises, are less successful in generating lessons where doing philosophy is effective. This observation stems from Peter’s lesson in [4], where a presentation was used, and Ramses’ lesson based on class discussion in the current study. This finding contradicts Higgins and Baumfield’s [35] plea for using general instead of domain-specific (such as philosophical) exercises.

A reviewer pointed out that Cognitive Load Theory (CLT) might explain this finding. Traditionally, CLT has focused on using instructional methods to decrease extraneous cognitive load, so that available cognitive resources can be fully devoted to learning [36]. The current view in instructional design is that authentic learning tasks are the driving force for complex learning [37]. Examples of authentic philosophical learning tasks include essay writing, a debate or Socratic dialogue, delivering a speech, and discussing dilemmas [38]. These ideas on CLT might explain why general exercises are less effective than philosophical exercises.

## Conclusions

The purpose of this article was to replicate our earlier research into doing philosophy effectively, and to deepen our understanding of what makes doing philosophy effective. We formulated the following research questions:

RQ1: Can the one-dimensional structure found in the earlier study by CA be replicated?

RQ2: Now that we have data from the earlier study and new data, can we obtain a better understanding of how teacher behavior leads to the level of doing philosophy?

For the first research question, the answer is positive. The one-dimensional structure can be replicated, where lessons are on a scale that ranges from lessons where doing philosophy is more effective to lessons where doing philosophy is less effective. Relationships with variables concerning teacher behavior are also very similar: In effective lessons we more often observe philosophical discussions (instead of classroom talk) and shared guidance (instead of loose or strong guidance). In both studies, the teachers of more effective lessons had more experience because of their educational training, in addition to the other minor differences mentioned in the discussion section.

In response to the second research question, based on both studies, we concluded that a philosophical discussion is a sufficient condition for identifying a lesson in which doing philosophy is effective. On the other hand, shared guidance is a necessary condition for an effective lesson, but it is not sufficient: in some lessons guidance was shared but doing philosophy was ineffective, whereas we found shared guidance in all effective lessons.

It is remarkable that we found such strong similarities between the two studies, given that both sample sizes were small. We classified most of the differences between the two studies as minor, and further replication of these studies, hopefully by other researchers, could shed light on these differences. We are currently conducting research in a similar but different field, religious education, where the methodology used in this study is also proving useful [39].

## Supporting information

S1 File Correspondence analysis, interpretation. (DOCX)

S2 File CA, Stability and analyses. (ZIP)

S3 File Super-indicator matrix taken from current study. (DOCX)

S4 File Super-indicator matrix and Burt matrix. (DOCX)

S5 File Super-indicator matrix taken from earlier study (Table 3 in [4]). (DOCX)
