# Supplementary material for: Doing philosophy effectively II: A replication and elaboration of student learning in classroom teaching
Source: PLoS One. 2018 Dec 3;13(12):e0208128. doi: 10.1371/journal.pone.0208128 (PMC6277092; doi:10.1371/journal.pone.0208128)
Supplement: S2 File — (ZIP) [file pone.0208128.s002.zip › CA, stability and analysis/Readme.docx]

First the analysis should be started with the syntax file SYNTAX NATASCHA 20181014 STABILITY.

In this syntax file the directory in the syntax file has to be changed so that the data can be found. I.e. the line CD has to be changed. The data are in NATASCHA 20170805 ENG.

When the file SYNTAX NATASCHA 20181014 STABILITY is run, for each analysis output files are created, for example SCORE1.

- One analysis is carried out for the full sample of 10 lessons with 13 variables. This yields the file SCORE1.sav.
- THEN 13 analyses are done, one for each variable that is left out of the analysis. For example, for the first analysis left out the file made is SCORE1min01.sav.
- Then an analysis is done where 3 variables are left out, this leads to the file SCORE1min3var.sav.
- Then analyses are done where 2 lessons are left out. This yields the files SCORE1minlesson12.sav, SCORE1minlesson110.sav, and SCORE1minlesson410.sav
- Then analysis are done where one lesson for John is let out, see SCOREminlesson1.sav and SCOREminlesson2.sav.

For all SCORExxx.sav files, we took out the quantification of the lessons by hand and placed them in the file STABILITY 20181014.sav. These quantifications are in the first 10, 8 or 9 elements of DIM1 in the SCORExxx.sav files. For the file STABILITY 20181014.sav CORRELATIONS are run, see file OUTPUT 20181014 STABILITY COR.spv. In this output the first column has the correlations reported in the text.
