# Supplementary material for: Doing philosophy effectively II: A replication and elaboration of student learning in classroom teaching
Source: PLoS One. 2018 Dec 3;13(12):e0208128. doi: 10.1371/journal.pone.0208128 (PMC6277092; doi:10.1371/journal.pone.0208128)
Supplement: S3 File — (DOCX) [file pone.0208128.s003.docx]

**S3 File. Super-indicator matrix of the meta-matrix in Table 1.**

|  | Approaches | | | Domains | | | | | Aim | | MA Phil. | | Exp. after  training | | St. grade | |  |  |
| --- | --- | --- | --- | --- | --- | --- | --- | --- | --- | --- | --- | --- | --- | --- | --- | --- | --- | --- |
|  | Jd | Ttf | Ctf | PA | ToK | Soc | Eth | PhSc | yes | no | yes | no | 1/10 | 11/15 | 10 | 11 |  |  |
| 1. | 0 | 0 | 3 | 0 | 0 | 1 | 0 | 0 | 0 | 1 | 1 | 0 | 1 | 0 | 1 | 0 |  |  |
| 2. | .5 | 0 | 2.5 | 0 | 0 | 1 | 0 | 0 | 0 | 1 | 1 | 0 | 1 | 0 | 1 | 0 |  |  |
| 3. | 0 | .5 | 2.5 | 1 | 0 | 0 | 0 | 0 | 1 | 0 | 1 | 0 | 1 | 0 | 1 | 0 |  |  |
| 4. | 1 | 2 | 0 | 0 | 0 | 0 | 1 | 0 | 1 | 0 | 1 | 0 | 0 | 1 | 1 | 0 |  |  |
| 5. | 0 | .5 | 2.5 | 0 | 1 | 0 | 0 | 0 | 1 | 0 | 1 | 0 | 1 | 0 | 0 | 1 |  |  |
| 6. | 0 | 1 | 2 | 0 | 1 | 0 | 0 | 0 | 1 | 0 | 1 | 0 | 0 | 1 | 0 | 1 |  |  |
| 7. | 0 | .5 | 2.5 | 0 | 0 | 1 | 0 | 0 | 1 | 0 | 0 | 1 | 0 | 1 | 1 | 0 |  |  |
| 8. | .5 | 0 | 2.5 | 0 | 0 | 0 | 1 | 0 | 0 | 1 | 1 | 0 | 1 | 0 | 1 | 0 |  |  |
| 9. | 0 | .5 | 2.5 | 1 | 0 | 0 | 0 | 0 | 1 | 0 | 1 | 0 | 0 | 1 | 1 | 0 |  |  |
| 10. | 0 | 2.5 | .5 | 0 | 0 | 0 | 0 | 1 | 1 | 0 | 1 | 0 | 0 | 1 | .5 | .5 |  |  |

*(continued)*

|  | Teaching styles | | Dialogue | | Guidance | | Number of Pearls | | | Duration (%) | | | Highest level | | Methods common concept formation | | | |
| --- | --- | --- | --- | --- | --- | --- | --- | --- | --- | --- | --- | --- | --- | --- | --- | --- | --- | --- |
|  | 1/2 | 3 | disc | crt | loose/  strong | shared | 123 | 4 | 567 | low/  mid | high | very  high | 4 | 5 | M1 | M2 | M3 | M4 |
| 1. | 1 | 0 | 0 | 1 | 1 | 0 | 1 | 0 | 0 | 1 | 0 | 0 | 1 | 0 | 0 | .69 | .31 | 0 |
| 2. | 1 | 0 | 0 | 1 | 0 | 1 | 1 | 0 | 0 | 1 | 0 | 0 | 1 | 0 | 0 | .40 | .19 | .41 |
| 3. | 1 | 0 | 0 | 1 | 0 | 1 | 0 | 0 | 1 | 1 | 0 | 0 | 1 | 0 | .54 | .27 | 0 | .19 |
| 4. | 1 | 0 | 1 | 0 | 0 | 1 | 0 | 1 | 0 | 0 | 0 | 1 | 0 | 1 | .14 | .67 | 0 | .19 |
| 5. | 0 | 1 | 0 | 1 | .5 | .5 | 1 | 0 | 0 | 1 | 0 | 0 | 1 | 0 | .42 | 0 | .24 | .34 |
| 6. | 0 | 1 | 0 | 1 | .5 | .5 | 0 | 1 | 0 | 1 | 0 | 0 | 1 | 0 | .30 | .20 | 0 | .50 |
| 7. | 1 | 0 | 0 | 1 | 0 | 1 | 0 | 1 | 0 | 0 | 0 | 1 | 1 | 0 | 0 | .31 | 0 | .69 |
| 8. | 1 | 0 | 0 | 1 | 1 | 0 | 0 | 1 | 0 | 0 | 1 | 0 | 1 | 0 | 0 | .67 | .08 | .26 |
| 9. | 1 | 0 | 1 | 0 | 0 | 1 | 1 | 0 | 0 | 0 | 1 | 0 | 0 | 1 | .72 | .28 | 0 | 0 |
| 10. | 1 | 0 | 1 | 0 | 0 | 1 | 0 | 0 | 1 | 0 | 1 | 0 | 0 | 1 | 0 | 0 | .09 | .91 |

For the abbreviations of the labels for the levels, see Table 1. Ten rows (lessons) and thirteen variables that have in total 34 levels.
