# Supplementary material for: Doing philosophy effectively II: A replication and elaboration of student learning in classroom teaching
Source: PLoS One. 2018 Dec 3;13(12):e0208128. doi: 10.1371/journal.pone.0208128 (PMC6277092; doi:10.1371/journal.pone.0208128)
Supplement: S4 File — (DOCX) [file pone.0208128.s004.docx]

**Supporting Information**

**S4 File, after S7 File in [2].**

**Super-indicator matrix and Burt matrix**

Here we discuss the general principles that are used for deriving the super-indicator matrix from the meta-matrix. The super-indicator matrix is the input matrix for doing a CA in SPSS. The discussion here relates to the super-indicator matrix from the *earlier* study [2], that was provided as Table 3 in [2]. In this paper we provide it again in Supplementary material S4 File.

In mathematics a so-called indicator variable is a variable having values 0 and 1, indicating for observations whether it has a certain level (category), then value is 1) or not (then value is zero. An indicator matrix is a matrix for a variable. Each variable has its own matrix. In this matrix the number of the columns equals the number of levels of a variable, and the number of rows is equal to the number of observations (here: 8, the number of lessons). For example, in the super-indicator matrix in S4 File the variable Aim has two levels, namely yes and no, and the indicator matrix therefore has 8 rows (lessons) and 2 columns (levels). The super-indicator matrix is a concatenation of the 13 indicator matrices (there are 13 variables) and is displayed in S4 File.

We used three ways of coding the data: simple indicator coding, fuzzy coding and equality constraints. Simple indicator coding is used for the variables Aim, MA philosophy, experience after training, Student grade, teaching styles, number of pearls, duration and highest level. For the number of pearls we made a split between 2 and 3 versus 4 to 6. Thus both levels contain 4 lessons and this provides stability to the CA solution. For the duration we made a split in the percentages displayed in the meta-matrix (see Table 2 in [2]).

The variables domains, guidance and methods of common concept formation are treated differently. For lesson 1 in the variable domains we see .5 for PA (philosophical anthropology) and Log (logic), showing that in this lessons two domains are covered. We choose two values adding up to 1. In the statistical literature of CA this is known as fuzzy coding [1]. In guidance, lesson 4 uses both loose as well as shared guidance. In methods of common concept formation we display calculated the total time in the lesson spent on pearls, and then derived the proportion of the time to methods 1, 2, 3 and 4; note that these proportions add up to 1 for each lesson.

In the first variable, approaches to doing philosophy, the levels Jd, Ttf and Ctf, we find for each lesson values adding up to three. This corresponds to the fact that in the meta-matrix this variable approaches was coded three times, namely for the design, for the execution and for the learning activity. These perspectives could each have been coded into a separate indicator matrix, yielding then three indicator matrices for approaches but by adding them up we force the quantifications for these three indicator matrices to be identical.

So far for the construction of the super-indicator matrix from the meta-matrix. In CA the super-indicator matrix is decomposed using a so-called generalized singular value decomposition (this is a tool used in mathematics that is closely related to an eigenvalue-eigenvector decomposition). For details see [1]. If we denote this super-indicator matrix by **G**, then the matrix multiplication **G^T^G** (where G**^T^** stands for “transpose of **G**”) gives us the so-called Burt matrix. A generalized singular value decomposition of **G** is equivalent to a generalized eigenvalue-eigenvector decomposition of **G^T^G**. These mathematical tools should not bother us here, it is importance to keep in mind that one way to understand CA is that it is based on the Burt matrix.

The Burt matrix is shown on the next page. It is a concatenation of all two-way tables or pairs of variables. It may be compared with a correlation matrix, where a correlation is then found for each pair of variables. Here instead of a correlation a two-way table is shown of categories of a variable by categories of another variable. On the diagonal we find sub-matrices of a variable with itself.

When both variables are coded with simple indicator coding, a two-way contingency table is obtained. For example, consider row 10-11 (yes, no for the third variable Aim) with columns 12-13 (yes, no for fourth variable Master in philosophy). This reveals that (Aim, MA Phil) = (yes,yes) happens 2 times, (Aim, MA Phil) = (no,no) happens 2 times, and (Aim, MA Phil) = (no,yes) happens 4 times. (Aim, MA Phil) = (yes,no) has frequency zero. So there is an order of (yes, yes) to (no,yes) to (no,no), suggesting that only if the teacher has a master in philosophy there is the possibility that the teacher subscribes to the aim of teaching philosophy by doing philosophy. A similar relation is found for reaching the highest level (lines 5 and 6 from below) and MA Phil: (Level, MA phil) = (5, yes) happens 3 times, (Level, MA phil) = (5, no) happens 3 times and (Level, MA phil) = (4, no) happens 2 times. Here there is an order of (5, yes), (5, no) and (4, no), suggesting that level 5 can only be reached if the teacher has a master in philosophy.

For variables that are not coded with simple indicator coding the interpretation is similar. For example, consider the two-way matrix of the first three lines (approaches Jd, Ttf and Ctf) and columns 10-11 (yes, no for the third variable Aim). In the super-indicator matrix we found a total count for Jd of 5. We see here in the Burt matrix that this 3 times goes together with Aim is yes, and 2 times with Aim is no. For Ttf the counts for Aim is (yes, no) are (2.5, 8) so Ttf has a much stronger relation with no, and for Ctf (yes, no) is (.5, 7) showing that Ctf almost always goes together with Aim is no.

Because CA of the super-indicator matrix is mathematically equivalent to CA of the Burt matrix, we conclude that CA displays all relations in the two-way tables simultaneously in a geometrical representation. Another way to put it is to start from the Burt matrix, and to say that CA helps in summarizing all information in the sub-matrices present in the Burt matrix.

**References**

[1] Gifi A (1990) Nonlinear Multivariate Analysis*.* New Jersey: Wiley.

[2] Kienstra N, Imants J, Karskens M, Van der Heijden PGM (2015) Doing Philosophy Effectively: Student Learning in Classroom Teaching. PLOS ONE 10, 9:e0137590. doi:10.1371/journal.pone.0137590.
