# Supplementary material for: Doing philosophy effectively II: A replication and elaboration of student learning in classroom teaching
Source: PLoS One. 2018 Dec 3;13(12):e0208128. doi: 10.1371/journal.pone.0208128 (PMC6277092; doi:10.1371/journal.pone.0208128)
Supplement: S5 File — (DOCX) [file pone.0208128.s005.docx]

S5 File. Super-indicator matrix taken from earlier study [1], Table 3.

Table 3. Super-indicator matrix of the meta-matrix in Table 2. For the abbreviations of the labels for the levels, see Table 2. Eight rows (lessons) and thirteen variables that have in total 37 levels.

|  | Approaches | | | Domains | | | | | | Aim | | MA Phil. | | Exp. after training | | | | St. grade | |
| --- | --- | --- | --- | --- | --- | --- | --- | --- | --- | --- | --- | --- | --- | --- | --- | --- | --- | --- | --- |
|  | Jd | Ttf | Ctf | PA | PhM | ToK | Soc | Eth | Log | yes | no | yes | no | 0 | 1/5 | 6/10 | 11/15 | 10 | 11/12 |
| 1. | 1 | 2 | 0 | .5 | 0 | 0 | 0 | 0 | .5 | 0 | 1 | 1 | 0 | 0 | 0 | 0 | 1 | 1 | 0 |
| 2. | 2 | 1 | 0 | 0 | 1 | 0 | 0 | 0 | 0 | 1 | 0 | 1 | 0 | 0 | 0 | 0 | 1 | 0 | 1 |
| 3. | 1 | 1.5 | .5 | 1 | 0 | 0 | 0 | 0 | 0 | 1 | 0 | 1 | 0 | 0 | 0 | 0 | 1 | 0 | 1 |
| 4. | 0 | 1.5 | 1.5 | 1 | 0 | 0 | 0 | 0 | 0 | 0 | 1 | 1 | 0 | 0 | 1 | 0 | 0 | 0 | 1 |
| 5. | 0 | 2 | 0 | 0 | 0 | 1 | 0 | 0 | 0 | 0 | 1 | 1 | 0 | 0 | 1 | 0 | 0 | 0 | 1 |
| 6. | 1 | 0 | 2 | 0 | 0 | 0 | 1 | 0 | 0 | 0 | 1 | 1 | 0 | 0 | 0 | 1 | 0 | 0 | 1 |
| 7. | 0 | 1 | 2 | 0 | 0 | 0 | 0 | 1 | 0 | 0 | 1 | 0 | 1 | 1 | 0 | 0 | 0 | 1 | 0 |
| 8. | 0 | 1.5 | 1.5 | 1 | 0 | 0 | 0 | 0 | 0 | 0 | 1 | 0 | 1 | 0 | 0 | 1 | 0 | 1 | 0 |
|  |  |  |  |  |  |  |  |  |  |  |  |  |  |  |  |  |  |  |  |
| *(continued)* | | | | | | | | | | | | | | | | | | | |
|  | Teaching styles | | Dialogue | | Guidance | | | # Pearls | | Duration (%) | | | Highest level | | Methods common concept formation | | | |  |
|  | 1 | 3 | disc | crt | loo | sh | str | 23 | 456 | lo | mid | high | 4 | 5 | M1 | M2 | M3 | M4 |  |
| 1. | 0 | 1 | 1 | 0 | 0 | 1 | 0 | 0 | 1 | 0 | 0 | 1 | 0 | 1 | .39 | .29 | 0 | .32 |  |
| 2. | 1 | 0 | 1 | 0 | 0 | 1 | 0 | 1 | 0 | 0 | 0 | 1 | 0 | 1 | 0 | .53 | 0 | .47 |  |
| 3. | 1 | 0 | 1 | 0 | 0 | 1 | 0 | 0 | 1 | 0 | 1 | 0 | 0 | 1 | 0 | .13 | .29 | .58 |  |
| 4. | 1 | 0 | 0 | 1 | .5 | .5 | 0 | 0 | 1 | 0 | 1 | 0 | 1 | 0 | .39 | .15 | .34 | .12 |  |
| 5. | 1 | 0 | 0 | 1 | 0 | 1 | 0 | 1 | 0 | 0 | 1 | 0 | 1 | 0 | 0 | 0 | .13 | .87 |  |
| 6. | 1 | 0 | 0 | 1 | 1 | 0 | 0 | 0 | 1 | 1 | 0 | 0 | 1 | 0 | .19 | .16 | .27 | .38 |  |
| 7. | 1 | 0 | 0 | 1 | 0 | 0 | 1 | 1 | 0 | 0 | 1 | 0 | 1 | 0 | .55 | .26 | 0 | .19 |  |
| 8. | 1 | 0 | 0 | 1 | 1 | 0 | 0 | 1 | 0 | 1 | 0 | 0 | 1 | 0 | 0 | 0 | 1 | 0 |  |

[1] Kienstra N, Imants J, Karskens M, Van der Heijden PGM (2015) Doing Philosophy Effectively: Student Learning in Classroom Teaching. PLOS ONE 10, 9:e0137590. doi:10.1371/journal.pone.0137590.
